# Somatic Mutation Profiling and Therapeutic Landscape of Breast Cancer in the MENA Region

**DOI:** 10.3390/cells14221791

**Published:** 2025-11-14

**Authors:** Dinesh Velayutham, Ramesh Elango, Sameera Rashid, Reem Al-Sarraf, Mohammed Akhtar, Khalid Ouararhni, Puthen Veettil Jithesh, Nehad M. Alajez

**Affiliations:** 1 College of Health & Life Sciences, Hamad Bin Khalifa University, Qatar Foundation, Doha P.O. Box 34110, Qatar; dineshvelayutham@qf.org.qa (D.V.);; 2 Translational Oncology Research Center, Qatar Biomedical Research Institute, Hamad Bin Khalifa University, Qatar Foundation, Doha P.O. Box 34110, Qatar; 3 Department of Laboratory Medicine and Pathology, Hamad Medical Corporation, Doha P.O. Box 34110, Qatar; 4 National Health Service, Royal Liverpool University, Liverpool L7 8XP, UK; 5 Genomics Core Facility, Hamad Bin Khalifa University, Qatar Foundation, Doha P.O. Box 34110, Qatar

**Keywords:** breast cancer, somatic mutations, somatic signature, MENA region, Arab, driver mutations, therapeutic implications

## Abstract

**Highlights:**

**What are the main findings?**

- Whole exome sequencing (WES) characterized the mutational landscape of breast cancer in MENA patients, revealing both known and novel potential driver mutations.
- Subtype-specific mutational signatures were identified.

**What is the implication of the main finding?**

- OncoKB annotation uncovered actionable variants, pointing to potential new therapeutic opportunities for breast cancer.
- These findings expand knowledge in an underrepresented MENA population, supporting precision oncology efforts.

**Abstract:**

Breast cancer remains a major global health challenge. Yet, genomic data from Middle Eastern and North African (MENA) populations are limited, restricting insights into disease drivers and therapeutic opportunities in this demographic. To address this gap, we performed whole-exome sequencing (WES) on 52 breast cancer samples, including 51 from the MENA region, to characterize somatic mutations and potential therapeutic targets. Across the cohort, 37,369 somatic variants matched entries in the COSMIC database, and driver prediction tools (BoostDM and OncodriveMUT) identified 2451 predicted driver mutations, including 648 known driver variants in genes such as TP53, PIK3CA, GATA3, PTEN, SF3B1, and KMT2C. In addition, 1803 novel predicted drivers were detected, many affecting DNA repair pathways, including homologous recombination (BRCA2, RAD51C), mismatch repair (MLH1, MSH2), and nucleotide excision repair (ERCC2, ERCC3), as well as regulators such as TP53 and ATM. Mutational signature analysis revealed a predominance of C>T substitutions and subtype-specific patterns, with SBS22 and SBS43 enriched in Luminal A tumors. Therapeutic annotation using OncoKB identified 223 actionable or likely oncogenic variants, highlighting potential targets for precision oncology. This study provides a comprehensive characterization of the breast cancer mutational landscape in MENA patients and offers a valuable resource for advancing genomic and therapeutic research in this demographic.

## 1. Introduction

Breast cancer is a highly heterogeneous disease characterized by genetic alterations that influence its biological behavior and response to therapy. The three major subtypes of breast cancer, based on receptor status, are estrogen receptor-positive (ER+), human epidermal growth factor receptor 2-positive (HER2+), and triple-negative breast cancer (TNBC). Each subtype is defined by unique genetic profiles that guide specific targeted therapies [1].

ER+ breast cancer accounts for approximately 70% of all breast cancer cases and is driven by estrogen signaling. Key genetic alterations in ER+ breast cancer include mutations in the PIK3CA gene, present in about 40% of cases, leading to activation of the PI3K/AKT/mTOR pathway [2]. Additionally, mutations in ESR1, the gene encoding the estrogen receptor, were found to contribute to resistance to endocrine therapies [3]. Targeted therapies for ER+ breast cancer include endocrine therapies such as tamoxifen and aromatase inhibitors, which block estrogen signaling, and CDK4/6 inhibitors such as palbociclib and ribociclib, which inhibit cell cycle progression when combined with endocrine therapy [4]. Alpelisib, a PI3K inhibitor, targets PIK3CA-mutant ER+ breast cancers and has proven effective in treating this patient group [5]. HER2+ breast cancer, representing about 15–20% of cases, is characterized by overexpression or amplification of the HER2 gene, leading to aggressive tumor growth. The HER2 gene encodes a tyrosine kinase receptor involved in cell proliferation and survival, with common genetic alterations including HER2 amplification and mutations in downstream signaling pathways, like PIK3CA [6]. Targeted therapies for HER2+ breast cancer include monoclonal antibodies such as trastuzumab and pertuzumab, which prevent receptor dimerization and signaling, and tyrosine kinase inhibitors like lapatinib and neratinib that inhibit the kinase activity of the HER2 receptor [7]. TNBC, which lacks expression of ER, progesterone receptors (PR), and HER2 amplifications, comprises about 10–15% of breast cancer cases and is genetically diverse. Frequent mutations in TNBC include TP53, which occur in over 80% of cases, and alterations in genes involved in DNA repair, such as BRCA1/2 [8,9]. Targeted therapies for TNBC include PARP inhibitors like olaparib and talazoparib, which target BRCA-mutated TNBC by inhibiting the PARP enzyme crucial for DNA repair [10]. Immune checkpoint inhibitors, such as pembrolizumab, have shown efficacy in TNBC, particularly in tumors with high PD-L1 expression [11].

Breast cancer is characterized by a highly heterogeneous somatic mutational landscape shaped by diverse mutational processes, including age-related, APOBEC-mediated, and homologous recombination deficiency–associated signatures. Large-scale genome sequencing studies, such as the analysis of 560 breast tumors identified 93 recurrently mutated driver genes, highlighting both the complexity and variability of the mutational background across molecular subtypes [12].

In the Arab region, breast cancer is the most common malignancy among women [13], exhibiting unique characteristics in terms of incidence, age at diagnosis, and genetic profiles compared to Western populations. Women in Arab countries tend to be diagnosed at younger ages and often present with more advanced stages of the disease [14]. Factors potentially contributing to these differences include genetic predispositions, environmental factors, and disparities in healthcare access. PTEN, a tumor suppressor regulating the PI3K-AKT pathway, is frequently lost in Middle Eastern breast cancers, particularly in triple-negative subtypes, and its inactivation is associated with aggressive tumor features and poor prognosis, highlighting its potential as a therapeutic target. [15]. In a meta-analysis of ~2500 patients across 44 studies focused on MENA breast cancer patients, TP53 mutations (≈23.8%) and PIK3CA mutations (≈10.2%) emerged as the most frequent somatic alterations, followed by ~mutations in BRCA1/2, ATM, ESR1, and PTEN [16]. These regional mutation patterns underscore the importance of context-specific genomic profiling and motivate our focus on driver and therapeutic mutation landscapes in a MENA breast cancer cohort.

BRCA1 and BRCA2 mutations, known risk factors for breast cancer globally, are also present among Arab women, though specific mutations and their frequencies can vary significantly. For example, a study identified frequent BRCA1 germline mutations such as c.1140dupG, c.4136_4137delCT, c.5095C>T, and c.5530delC in Arab breast cancer patients, suggesting potential mutations within this population [17]. A study from Saudi Arabia additionally highlighted significant germline mutations in the BRCA1 and BRCA2 genes in high-risk breast cancer patients, essential for understanding breast cancer susceptibility in Arab women [18]. Other notable genetic variants implicated in breast cancer among Arab women include germline polymorphism in the TP53 gene [15,19].

Advancements in genomic technologies have facilitated more comprehensive studies of breast cancer genetics in the Arab region, reflecting a unique landscape shaped by historical migrations and population mixing. In Qatar, Saad et al. analyzed genetic variations across different ancestry groups. The study revealed significant differences in polygenic risk scores for common cancers, including breast cancer, among these groups, where those of Persian origin exhibited a higher frequency of BRCA1/BRCA2 pathogenic variants compared to those of Arabian Peninsula origin, highlighting the genetic heterogeneity within the population [20].

The unique genetic profile of breast cancer in the Arab population, potentially influenced by factors such as consanguinity and population-specific mutations, highlights the importance of region-specific genetic research. In this study, we conducted a comprehensive mutational analysis of sporadic breast cancer from Qatar, identifying both known and novel mutations. By integrating our findings with the Catalogue of Somatic Mutations in Cancer (COSMIC) [21], Cancer Genome Interpreter [22], and OncoKB databases [23], we revealed the mutational landscape of breast cancer from this demographic. Our analysis provides valuable insights into potential therapeutic implications for this population, setting the foundation for future research focused on somatic mutations in breast cancer across the MENA region.

## 2. Materials and Methods

### 2.1. Patient Characteristics

Archived Formalin-Fixed Paraffin-Embedded (FFPE) tissue samples were obtained from fifty-two patients with breast cancer from the Department of Laboratory Medicine and Pathology (DLMP) at Hamad Medical Corporation (HMC), Doha, Qatar. Clinical and survival data were retrieved from electronic medical records. The study included female patients with invasive breast carcinoma who had undergone surgical excision of the primary tumor at HMC. Exclusions comprised cases of post-neoadjuvant chemotherapy, in situ malignancies, males, recurrent breast cancer, sarcomas, and metastatic carcinomas from non-breast primary tumors. Fifty-one patients were designated as Middle Eastern and North African (MENA). PAM50 classification (Basal, Luminal A, Luminal B, or HER2) was conducted as we described before [24]. Based on disease recurrence, the samples are classified into relapse and remission. Hormone signature-based groups include HER2+, HER2−, ER+ and ER−. Detailed patient and tumor characteristics are provided in Table 1.

### 2.2. Genomic DNA (gDNA) Extraction

gDNA extraction from FFPE core punches was performed using the AllPrep DNA/RNA FFPE kit (Qiagen, Hilden, Germany) with slight modifications. Initially, core punches (<35 mg) were transferred into a mortar containing liquid nitrogen and ground thoroughly using a pestle. The samples were then deparaffinized by incubating in xylene for 3 min at 50 °C, followed by centrifugation and two washes in 100% ethanol. The resulting pellets were vacuum-dried. Proteins were degraded using proteinase K enzyme, followed by a 15 min incubation and then centrifugation to collect the pellet for DNA extraction and supernatant for RNA extraction. After centrifugation, the pellet was resuspended in ATL Buffer with proteinase K and mixed by vortex. Digestion was carried out at 56 °C for 1 h, followed by reverse formaldehyde crosslinking reactions at 90 °C for 2 h without agitation.

The samples were cooled to room temperature, followed by RNAse treatment. Subsequently, 200 μL Buffer AL and 200 μL of 100% ethanol were added and mixed thoroughly by vortex. The entire lysate was transferred to a QIAamp MinElute spin column and centrifuged at 8000× *g* for 15 s. The column was washed sequentially by adding 700 μL Buffers AW1, AW2, and 100% ethanol. Finally, the QIAamp MinElute spin column was centrifuged at full speed for 5 min to remove residual ethanol. DNA was eluted in TAE elution buffer. The concentration and purity of extracted DNA were measured using Nanodrop 2000 (Thermo Scientific, Wilmington, NC, USA), and DNA was stored at −80 °C until further use.

### 2.3. Whole Exome Sequencing (WES)

Extracted gDNA was used as input for library preparation using the Agilent SureSelectXT kit from Agilent, Santa Clara, CA, USA, following the manufacturer’s protocol. For samples with high DNA integrity (high DIN), 200 ng of gDNA was mechanically fragmented using a Covaris E220 ultrasonicator; for samples with low DIN (highly fragmented FFPE DNA), they were used directly without additional shearing, as recommended for FFPE-derived DNA. The sheared DNA was purified with AMPure XP magnetic beads and then subjected to End-repair, followed by adenylation, and then ligated to the SureSelect DNA adapter. The ligated DNA was purified with AMPure XP magnetic beads. The library DNA was amplified by PCR and then purified. The library was subjected to hybridization with the specific biotin probes and then captured by streptavidin beads. After amplification by PCR using the specific index, the quality of the library generated was checked on an Agilent 2100 Bioanalyzer system and quantified using the Qubit system. Library preparation and Bioanalyzer analysis confirmed successful production of the desired fragment size, with an average insert of ~280–300 bp (Figure S1). Libraries that passed quality control were pooled and sequenced on a NextSeq2000 system at a minimum of 50 million paired-end reads (2 × 100 bp) per sample.

### 2.4. WES Data Analysis and Variant Calling

The raw sequence data underwent quality trimming for base quality score Q30 and adapter sequence and alignment to the hg19 reference genome using BWA-MEM as described before [25]. Comprehensive sequencing quality control was performed for all 52 WES samples. Adapter trimming and base quality filtering were conducted using Trimmomatic [26], retaining reads with Phred scores ≥ Q30. Quality assessment showed >95% of bases with Q30 or higher, and Picard-based post-alignment metrics indicated 90–95% of reads successfully aligned to the hg19 reference genome. The mean on-target coverage across captured regions was approximately 100×, with >90% of target bases covered at ≥20×, consistent with the expected performance for Agilent SureSelect XT–based exome sequencing. Somatic variant calling was performed in tumor-only mode using VarScan2 [27], while known germline variants were subtracted out to isolate somatic cells using UNMASC, as previously described previously [28,29]. Pileup files were generated from processed BAM files using SAMtools (v1.17) with a minimum mapping quality of 30 and a base-quality threshold of 20 to minimize sequencing noise. VarScan2 parameters included a minimum variant allele frequency (VAF) of 0.05, minimum coverage of 10×, and strand-filtering enabled, allowing the detection of both high and low-frequency somatic variants that may arise in heterogeneous tumor samples. Common germline polymorphisms were filtered using population databases including dbSNP (build 155), 1000 Genomes Phase 3, gnomAD-Genomes (v3.1), and ExAC (v0.3).”

Further analysis involved the prediction of driver genes using BoostDM/OncodriveMUT [22,30], within the ‘CancerGenomeInterpreter’ framework. R packages, MutationalPattern (v1.1.0), SigProfilerMatrixGenerator (v1.2.20), and SigProfilerAssignment (v0.1.0) were employed to extract somatic signatures [31,32,33], which were then compared with COSMIC (v3.4) single-base substitution (SBS) signatures. Therapeutic implications were inferred from OncoKB (v4.0) [23], a comprehensive knowledge base followed by annotation utilizing VEP, SNPeff, and OpenCravat [34,35,36].

### 2.5. Enrichment Analysis and Data Visualization

KEGG pathway enrichment analysis for COSMIC-annotated and novel predicted driver genes was performed using STRING (v11) as described before [37,38]. Only genes harboring predicted driver mutations were included in the analysis to identify biological pathways enriched among functionally relevant mutations. Gene sets were uploaded to STRING to identify significantly enriched pathways based on known and predicted protein–protein interactions. Significance was determined after Benjamini–Hochberg false discovery rate (FDR) correction, and pathways with an adjusted *p*-value < 0.05 were considered significant. This approach allowed functional interpretation of both previously reported and novel driver mutations in the context of cancer-related biological processes. SNP density, treemap, and alluvial plots were created using SRplot [39].

## 3. Results

### 3.1. Mutation Burden Analysis

**Figure 1:**
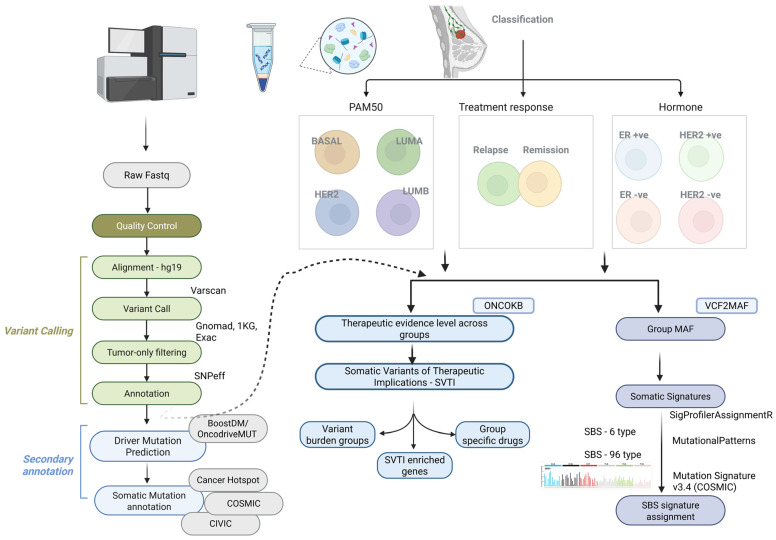
Experimental workflow for whole-exome sequencing (WES) data analysis, variant calling, and their therapeutic implications. Raw sequence data were quality-trimmed (Q30) and aligned to the hg19 reference genome using BWA-MEM. Somatic variants were called with Varscan2, and germline variants were excluded to retain somatic mutations. Driver gene prediction was performed using BoostDM/OncodriveMUT in the CancerGenomeInterpreter framework. Somatic signatures were extracted with R packages and compared to COSMIC SBS signatures. Therapeutic implications were derived using OncoKB, and samples were classified by PAM50 (Basal, LumA, LumB, HER2) and ER/HER2 status (ER+; ER−; HER2+; HER2−), as well as relapse vs. non-relapse. Annotations were performed using VEP, SnpEff, and OpenCRAVAT.

Figure 1 outlines the experimental workflow for WES data analysis, variant calling, and therapeutic annotations.

To identify potential driver mutations and mutations commonly reported in breast and other cancers, somatic variants from this study were compared across catalog databases. Using the COSMIC database, we identified 37,369 somatic variants that matched existing entries (Table S1).

**Figure 2:**
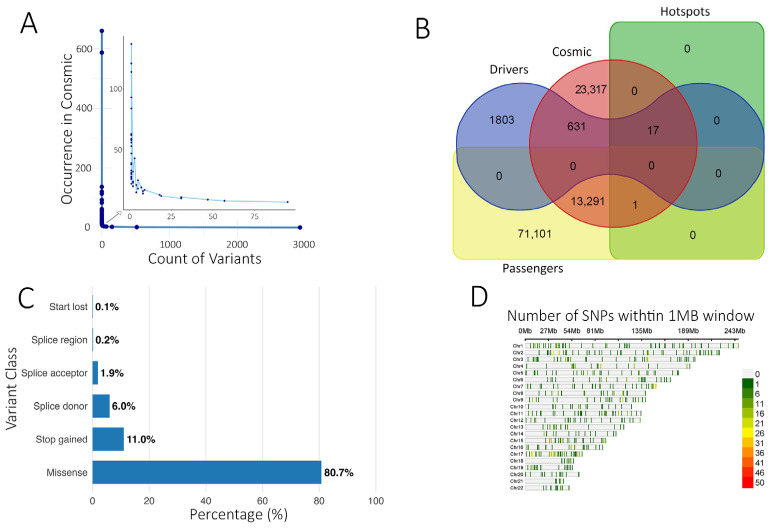
Analysis of somatic variant annotation. (**A**) Number of somatic variants identified in this study (x-axis) and their occurrence in COSMIC (y-axis). A Few variants have been identified more than twice, indicating a mutational hot spot (zoomed area). (**B**) Venn diagram illustrating the overlap between predicted driver and passenger variants in the current study with the COSMIC and Hotspots databases. (**C**) Functional class distribution of driver mutations predicted from the current study. (**D**) SNP plot showing the chromosomal distribution of identified driver variants within a 1 MB window. The color scale represents SNP density.

Of these, 8206 variants were observed at least twice in the database, indicating potential recurrence across cohorts. Five variants appeared more than 100 times (SF3B1:p.Lys700Glu, TP53:p.Cys141Tyr, TP53:p.Val173Leu, KMT2C:c.850-30A>G, and TP53:p.Tyr220Cys), while the majority were singletons (Table S1, Figure 2A).

Within the ‘Cancer hotspots database’ [40], 18 variants were also present in our cohort, mainly missense, with stop-gained variants observed in RNF43 and EPHA3. TP53 harbored 8 predominant missense variants within these hotspots. Three of the 18 variants were also reported in CIVIC and COSMIC, including VHL:p.Arg161Gln (adrenal/kidney), SF3B1:p.Lys700Glu, and TP53:p.Tyr220Cys (breast cancer). The SF3B1:p.Lys700Glu variant was predicted as a driver by the Cancer Genome Interpreter (Table S1). Using BoostDM and OncodriveMUT, we predicted 2451 potential driver mutations alongside 84,451 predicted passenger mutations (Figure 2B, Tables S2 and S3). Predicted driver variants were predominantly missense (80.7%), followed by stop-gained (11.0%), splice donor (6.0%), splice acceptor (1.87%), splice region (0.2%), and start lost (0.12%) (Figure 2C). Chromosomal distribution is shown in Figure 2D.

**Figure 3:**
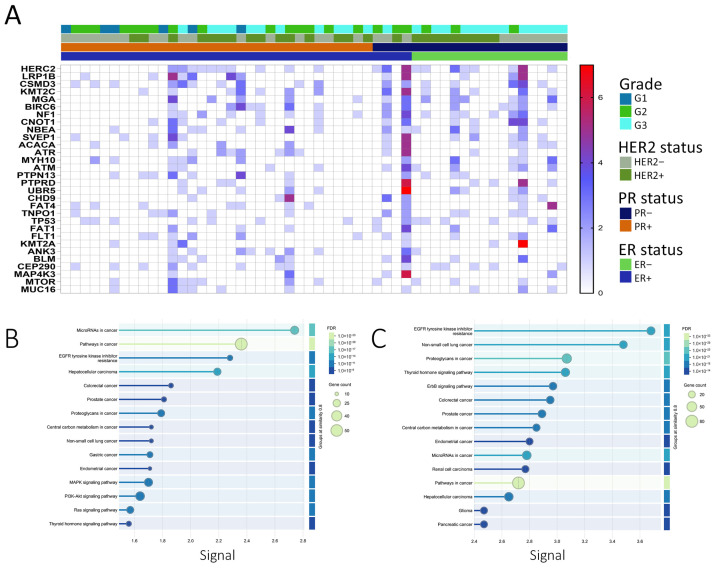
Mutational landscape and pathway enrichment of breast cancer samples from the MENA cohort. (**A**) Heatmap depicting the top 30 frequently mutated predicted driver genes from the current study, shown in relation to ER, PR, and HER2 status, as well as tumor grade. The color scale represents the number of mutations in each gene. (**B**) KEGG pathway enrichment analysis of genes harboring COSMIC-annotated driver mutations identified in the current study, performed using the STRING database (v11). Only predicted driver genes were included to determine biological pathways significantly enriched among functionally relevant mutations. Pathways were ranked by significance after Benjamini–Hochberg FDR correction (adjusted *p* < 0.05), and the top 15 enriched KEGG pathways are shown. (**C**) KEGG pathway enrichment analysis of genes harboring novel predicted driver mutations identified in this study, conducted as described above. The top 15 significantly enriched KEGG categories after multiple testing correction are depicted.

The top 30 genes with the highest predicted driver mutation frequency are presented as a heatmap (Figure 3A). Of the predicted driver mutations, 648 matched COSMIC entries, while 1803 were classified as novel predicted drivers (Figure 2B). KEGG enrichment of COSMIC-annotated drivers revealed involvement in PI3K-Akt, EGFR tyrosine kinase inhibitor resistance, and MAPK signaling pathways (Figure 3B). Enrichment analysis of all predicted drivers showed associations with cancer-related pathways, including Pathways in cancer, Proteoglycans in cancer, PI3K-Akt, MAPK, p53 signaling, platinum drug resistance, and multiple site-specific cancers (Figure 3C, Tables S4 and S5). Several mutated genes were implicated in DNA damage response and repair, including homologous recombination (BRCA2, RAD51C, PALB2, RAD54L), mismatch repair (MLH1, MSH2, MSH3, PMS1, PMS2, MLH3), nucleotide excision repair (XPA, XPC, ERCC2, ERCC3, RAD23B, BIVM-ERCC5), and base excision repair (FEN1, POLG), along with regulators such as TP53, ATM, ATR, WRN, MRE11, RAD50, and TP53BP1. These findings suggest potential mechanistic links between impaired genome maintenance and tumor progression, although functional validation is needed.

### 3.2. Somatic Signature Analysis

**Figure 4:**
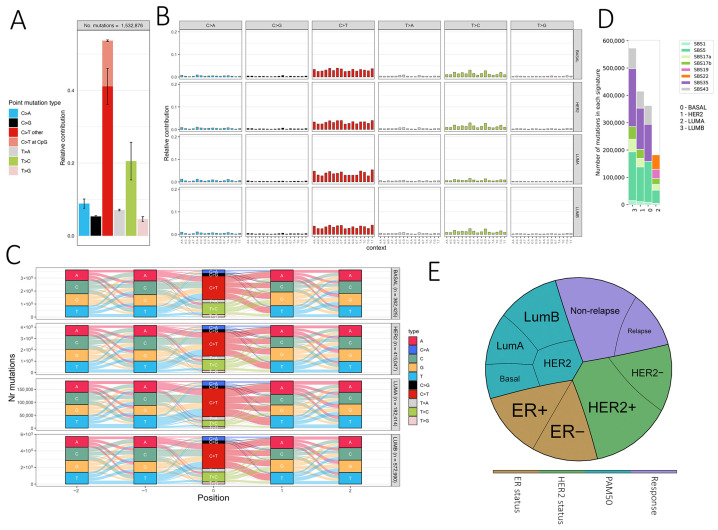
Mutational signatures and therapeutic implications of somatic variants in breast cancer. (**A**) Bar plot showing the relative frequency of six single base substitution types (SBS type-6), with C>T transitions at CpG sites highlighted. (**B**) SBS type-96 mutational profiles, displaying the trinucleotide context of mutations. Each panel shows the relative contribution of mutation types across samples. (**C**) River plots representing sequence context (±2 bp) of somatic mutations across PAM50 breast cancer subtypes (Basal, LumA, LumB, HER2). Mutation types are color-coded, and the y-axis indicates the number of mutations. (**D**) Distribution of COSMIC SBS signatures across PAM50 subtypes, with the number of mutations associated with each signature shown on the y-axis. (**E**) Pie chart summarizing the distribution of ER/HER2 status, PAM50 subtypes, and relapse outcome across the cohort.

Somatic mutation signatures can provide insights into underlying mutational processes [41]. In our cohort, SBS type-6 mutations predominantly showed C>T transitions followed by T>C (Figure 4A), consistent with methylated cytosine deamination. PAM50 subtypes exhibited similar SBS profiles, though LumA displayed a modest reduction in T>C substitutions (Figure 4B). Analysis in the extended 96-context framework revealed seven SBS signatures (SBS1, SBS5, SBS17a/b, SBS19, SBS22, SBS35, SBS43) (Figure 4C). Clock-like SBS1 and SBS5 were broadly present, while other signatures varied by subtype, e.g., LumA showed SBS19 and SBS22, Basal lacked SBS17a/b, and SBS35 was absent in LumA. These patterns are consistent with previously reported breast cancer signatures but should be interpreted cautiously given the cohort size and potential FFPE artifacts.

### 3.3. Therapeutic Implications of Somatic Variants

**Figure 5:**
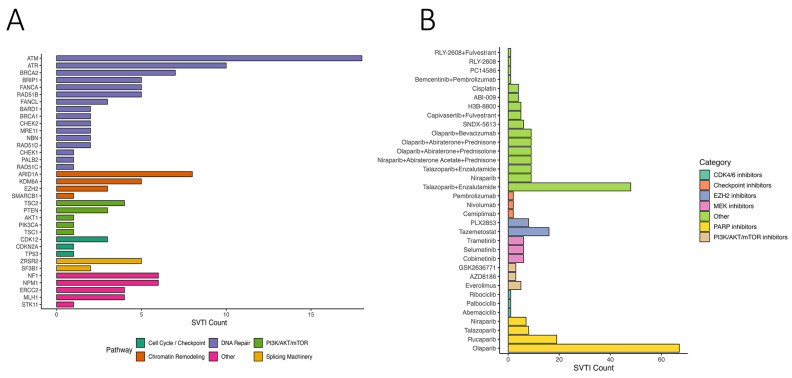
Therapeutic relevance of somatic variants of therapeutic importance (SVTIs) in breast cancer. (**A**) Bar chart illustrating the number of somatic variants of therapeutic importance (SVTIs) detected in key genes, grouped by functional pathways including DNA repair, PI3K/AKT/mTOR, chromatin remodeling, splicing, and cell cycle regulation. (**B**) Therapeutic annotations of SVTIs, displaying the number of variants linked to specific drugs. Drugs are categorized by mechanism of action, including PARP, CDK4/6, PI3K/AKT/mTOR, and checkpoint inhibitors.

OncoKB annotation identified 223 genes with variants classified as “Oncogenic” or “Likely Oncogenic” (LO) across at least one group (Figure S2A). Nineteen genes harbored LO variants in all four groups, including LRP1B, KMT2C, ATM, and TP53 (Figure S2B). We further identified predicted actionable variants (SVTIs) and categorized them by pathway: DNA repair, PI3K/AKT/mTOR signaling, cell cycle/checkpoint regulation, chromatin remodeling, splicing, and others (Figure 5A). DNA repair genes accounted for the largest fraction, suggesting potential targetable vulnerabilities.

Mapping SVTIs to therapeutic agents revealed drugs/classes potentially targeting these variants, including PARP inhibitors (olaparib, rucaparib), PI3K/AKT/mTOR inhibitors, checkpoint inhibitors, CDK4/6 inhibitors, EZH2 inhibitors, and MEK inhibitors (Figure 5B). PARP inhibitors showed the highest predicted coverage across SVTIs, consistent with prior evidence in DNA repair-deficient tumors. The combination of Talazoparib and Enzalutamide was also frequently predicted. These findings indicate potential therapeutic relevance, which requires validation in larger cohorts.

## 4. Discussion

Genetic insights from this study are crucial for advancing personalized medicine approaches tailored to breast cancer patients from the MENA region. Understanding the unique genetic landscape of breast cancer in this region can enhance screening programs, risk assessment models, and targeted therapies. This study provides a map of somatic variation in breast cancer patients from Qatar, representing the broader MENA region. We predicted 2451 potential driver mutations, of which 648 matched entries in the COSMIC database. Interestingly, the novel driver mutations identified in the current study involve genes with known roles in different cancer types, including breast cancer [42]. Those include mutations in PALB2, TP53, BRCA2, ATM, ATR, PIK3CA, and many other genes, thus uncovering the mutational signature for breast cancer patients from this region. Further investigations revealed 18 cancer hotspots [40] mutations in EPHA3, FUBP1, GATA3, MAP2K4, PTEN, RNF43, SF3B1, SMAD2, TP53, and VHL. Eight variants were seen in TP53, two variants in SF3B1, while the remaining genes harbored single cancer hotspot mutations. Exploring the COSMIC database revealed several SBS signatures in relation to breast cancer subtypes and treatment outcome. SBS1 is a cell division/mitotic clock-like signature observed in many types of cancers, including breast cancer, resulting from the spontaneous deamination of 5-methyl-cytosine [43,44]. SBS5, another clock-like signature, has been reported in breast cancer [45]. The LumA group exhibits a distinct SBS22 signature closely associated with aristolochic acid exposure, particularly evident in cancer samples with confirmed consumption of herbal products containing aristolochic acids. Notably, we have also identified signatures of unknown etiology, such as SBS17a and b and SBS19, the understanding of which could significantly contribute to our understanding of breast cancer development. Predicted therapeutic implications suggest DNA repair-related variants may represent potentially targetable vulnerabilities, with PARP inhibitors showing the highest predicted coverage. This finding aligns with prior evidence supporting the efficacy of PARP inhibition in tumors with defective DNA repair pathways, such as those harboring BRCA1/2 mutations. The data suggest that PARP inhibitors may offer therapeutic benefit beyond germline BRCA-mutant cases, extending to a broader group of patients with somatic alterations in DNA repair genes. SVTI profiling also highlighted alterations in PI3K/AKT/mTOR, cell cycle, chromatin remodeling, and splicing pathways. These observations should be interpreted as exploratory, given the cohort size and the lack of matched normals, but they provide hypotheses for future precision oncology studies.

Interestingly, Splicing Factor 3b Subunit 1 (SF3B1) [46] identified in one HER2− patient. Mutations in RNA splicing machinery components are prevalent across various hematologic malignancies and solid tumors, indicating the crucial role of aberrant splicing in cancer development. Among these, heterozygous somatic hotspot mutations in the spliceosomal component, SF3B1, are particularly common. These mutations are highly frequent in leukemia and, though less common, are also found in solid tumors such as cutaneous melanoma (4%), breast (2%), pancreatic (2%), lung (2%), and prostate cancer (1%) [47]. Notably, hotspot SF3B1 mutations were linked to poor outcomes in breast cancer patients [48]. Despite the modest sample size, our data align with global SF3B1 mutation rates in breast cancer. Analysis of 13,146 breast cancer patients shows Lys700Glu as the most predominant mutation (Figure S3). Enrichment of high-evidence SVTIs in non-relapse patients versus relapse suggests that patients with better outcomes may harbor more predicted actionable variants. These associations require validation in larger, longitudinal studies.

## 5. Conclusions

This study provides comprehensive somatic mutational profiling of breast cancer in patients from Qatar, offering valuable insights into the broader MENA genetic landscape. By integrating whole exome sequencing, driver prediction, mutational signature analysis, and preliminary therapeutic annotation, we identified known and novel driver mutations, many affecting DNA repair and oncogenic pathways, and observed subtype-specific somatic signatures. Potential therapeutic targets, particularly those linked to DNA repair, were highlighted, though their clinical relevance remains exploratory. Several limitations must be acknowledged. The modest cohort size (*n* = 52, including 51 MENA cases) restricts statistical power, though the availability of well-annotated specimens adds value. The absence of matched normal tissue prevented definitive somatic–germline distinction; despite stringent filtering with gnomAD and dbSNP, misclassification cannot be excluded. All samples were FFPE-derived, raising the possibility of artifacts, although mutational signatures suggested minimal bias. We acknowledge that, as a study limitation, we did not assess the potential impact of FFPE block age on mutation detection. Furthermore, the lack of genomic ancestry profiling limits generalizability, as population stratification may influence mutational patterns. Finally, the novel drivers identified here require validation in larger, longitudinal, and ancestrally diverse cohorts to establish biological and clinical relevance. Despite these limitations, this work lays a foundation for advancing region-specific precision oncology in the MENA region.

## Figures and Tables

**Table 1:** Clinical characteristics of the study cohort.

Ethnicity	
MENA	51 (98%)
Non-MENA	1 (2%)
Age (Median)	23–72 (44.5)
PAM50 subtype	
LumA	12 (13%)
LumB	20 (20%)
HER2	11 (29%)
Basal	9 (38%)
ER status	
ER+	36 (69%)
ER−	16 (31%)
HER2 status	
HER2+	25 (48%)
HER2−	27 (42%)
Clinical outcomes	
Relapse	16 (31%)
Non-relapse	32 (61%)
NA	4 (8%)

## Data Availability

Processed data are provided in the Supplementary Materials. Additional data are available from the corresponding author upon reasonable request.

## Supplementary Materials

The following supporting information can be downloaded at: https://www.mdpi.com/article/10.3390/cells14221791/s1. Figure S1: Representative Agilent 2100 Bioanalyzer electropherogram traces of whole-exome sequencing (WES) libraries prepared from FFPE-derived breast cancer DNA using the Agilent SureSelectXT workflow; Figure S2: Somatic signature analysis in relation to treatment groups; Figure S3: Analysis of SF3B1 mutations in 11,346 breast cancer patients from cBioPortal; Table S1: Annotated COSMIC variants identified in this study that are mutually present in CancerHotspots, CGI, and CIVIC, with genomic coordinates based on the hg38 assembly; Table S2: Predicted driver mutations identified using machine learning algorithms, annotated as “novel” or “known,” with genomic coordinates mapped to both hg19 and hg38 assemblies; Table S3: Predicted passenger mutations identified using machine learning algorithms, annotated as “novel” or “known,” with genomic coordinates mapped to both hg19 and hg38 assemblies; Table S4: Novel driver mutations predicted in this study using machine learning algorithms and their counts per gene; Table S5: Enriched functional categories among predicted novel driver mutated genes identified via STRING PPI network analysis.

## Abbreviations

The following abbreviations are used in this manuscript:

ER	Estrogen Receptor
HER2	Human Epidermal Growth Factor Receptor 2
TNBC	Triple-Negative Breast Cancer
PR	Progesterone Receptor
PI3K	Phosphoinositide 3-Kinase
AKT	Protein Kinase B
mTOR	Mechanistic Target of Rapamycin
CDK4/6	Cyclin-Dependent Kinases 4 and 6
FFPE	Formalin-Fixed Paraffin-Embedded
DLMP	Department of Laboratory Medicine and Pathology
HMC	Hamad Medical Corporation
MENA	Middle East and North Africa
WES	Whole Exome Sequencing
gDNA	Genomic DNA
BWA-MEM	Burrows-Wheeler Aligner Maximal Exact Match
PAM50	Prediction Analysis of Microarray 50
VEP	Variant Effect Predictor
SNPeff	SNP Effect Predictor
KEGG	Kyoto Encyclopedia of Genes and Genomes
COSMIC	Catalogue of Somatic Mutations in Cancer
SBS	Single Base Substitution
GO	Gene Ontology
UNMASC	Unmatched Normals and Matched Analysis of Somatic Calls
OncoKB	Oncology Knowledge Base
CIVIC	Clinical Interpretation of Variants in Cancer
STRING	Search Tool for the Retrieval of Interacting Genes/Proteins
SRplot	Shiny R Plot
