# Supplementary material for: Somatic Mutation Profiling and Therapeutic Landscape of Breast Cancer in the MENA Region
Source: Cells. 2025 Nov 14;14(22):1791. doi: 10.3390/cells14221791 (PMC12651733; doi:10.3390/cells14221791)
Supplement: Supplementary file 1 [file cells-14-01791-s001.zip › cells-3910392-supplementary/Figure S1.pdf]

Assay Class: High Sensitivity DNA Assay
Data Path: D:\...gh Sensitivity DNA Assay_DE13806056.xad

Electrophoresis File Run Summary

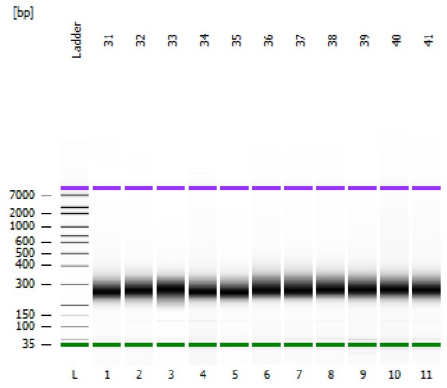

Instrument Information:

Instrument Name: DE13806056 Firmware: C.01.069

Serial#: DE13806056 Type: G2938C

Assay Information:

Assay Origin Path: C:\Program Files (x86)\Agilent\2100 bioanalyzer\2100 expert\assays\dsDNA\High Sensitivity DNA.xsy

Assay Class: High Sensitivity DNA Assay

Version: 1.03

Chip Information:

Chip Lot #:

Reagent Kit Lot #:

Chip Comments:

31

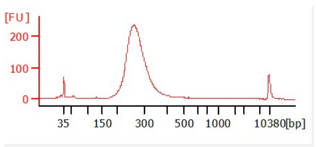

32

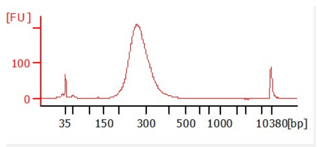

33

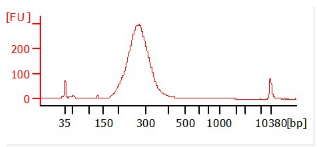

34

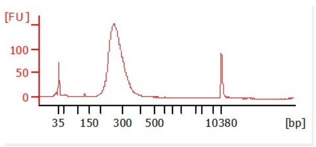

35

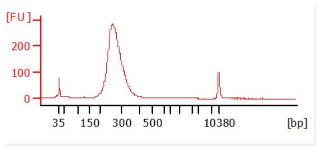

36

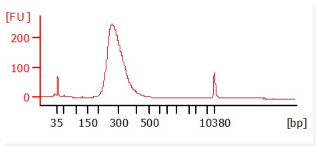

37

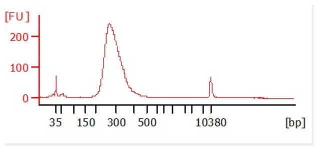

38

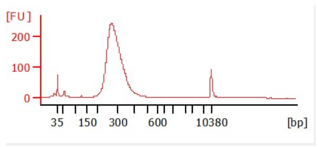

39

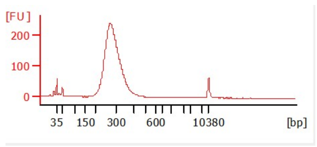

40

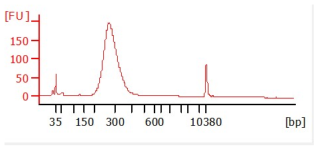

41

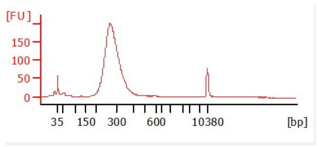

Figure S1. Representative Agilent 2100 Bioanalyzer electropherogram of whole-exome sequencing (WES) libraries prepared from FFPE-derived breast cancer DNA using the Agilent SureSelectXT workflow. The High Sensitivity DNA assay shows a predominant peak at approximately 280–300 bp, indicating the successful generation of libraries with the desired fragment size distribution.
