# Supplementary material for: Somatic Mutation Profiling and Therapeutic Landscape of Breast Cancer in the MENA Region
Source: Cells. 2025 Nov 14;14(22):1791. doi: 10.3390/cells14221791 (PMC12651733; doi:10.3390/cells14221791)
Supplement: Supplementary file 1 [file cells-14-01791-s001.zip › cells-3910392-supplementary/Figure S2.pdf]

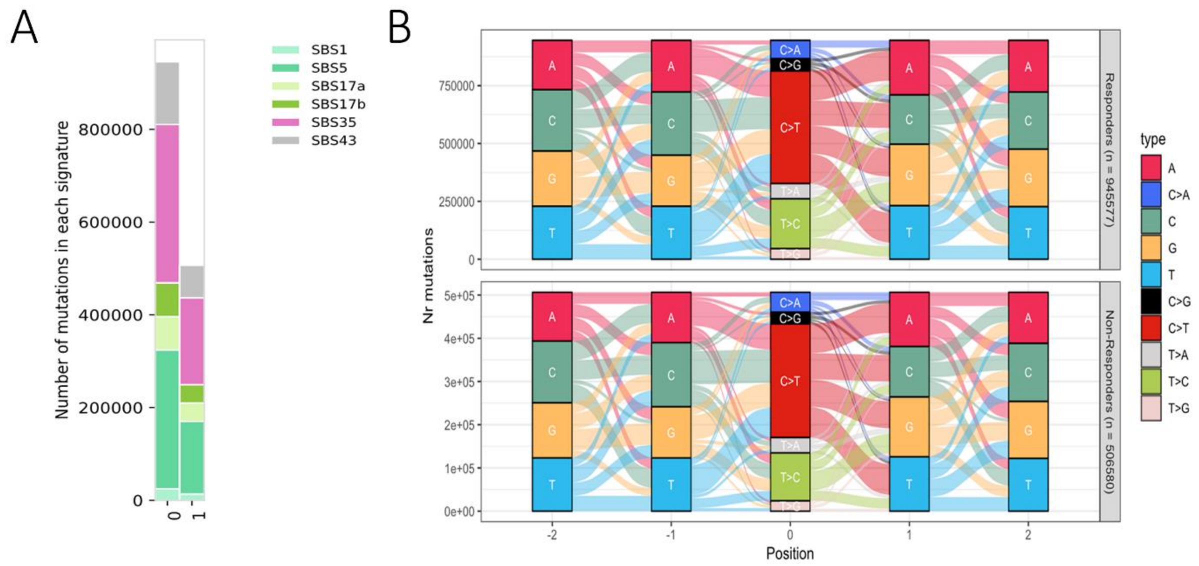

Figure S2. Somatic signature analysis in relation to treatment groups. (A) SBS Somatic signatures in relapse[1] and non-relapse groups **[0]. (B)** 96-type somatic signatures in relapse and non-relapse groups.
