# Supplementary material for: Somatic Mutation Profiling and Therapeutic Landscape of Breast Cancer in the MENA Region
Source: Cells. 2025 Nov 14;14(22):1791. doi: 10.3390/cells14221791 (PMC12651733; doi:10.3390/cells14221791)
Supplement: Supplementary file 1 [file cells-14-01791-s001.zip › cells-3910392-supplementary/Figure S3.pdf]

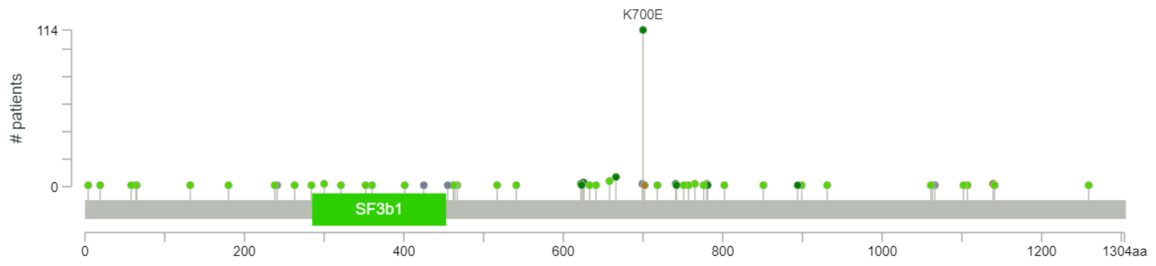


**Figure S3. Analysis of SF3B1 mutations in 11,346 breast cancer patients from cBioPortal.** The figure highlights the K700E mutation as the most commonly observed mutation.
